# Observations on Sloughing Sores

**Published:** 1827-10

**Authors:** G. G. Babington

**Affiliations:** Surgeon to the Lock Hospital.


THE LONDON
### Medical and Physical Journal.

No. 344, VOL. LVIII.] OCTOBER, 1827. [No. 16, *New Series*.

For many fortunate discoveries in medicine, and for the detection of numerous errors, the world is indebted to the rapid circulation of Monthly Journals; and there never existed any work, to which the Faculty, in Europe and America, were under deeper obligations, than to the Medical and Physical Journal of London, now forming a long, but an invaluable, series.—RUSH.

### ORIGINAL PAPERS,

AND

### CASES OBTAINED FROM PUBLIC INSTITUTIONS AND OTHER AUTHENTIC SOURCES.

### SLOUGHING SORES.

*Observations on Sloughing Sores.* By G. G. BABINGTON, Esq. Surgeon to the LOCK HOSPITAL.

SOME months since there appeared in the London Medical and Physical Journal several cases of sloughing sores in the genitals, collected from the different hospitals in London. A few of these had occurred under my care in the Lock Hospital, and were calculated to illustrate the different modes of treatment, which I believe to be adapted to their different varieties. As the principles of distinction, by which I was guided in the choice of the remedies, were not detailed on that occasion, and appear to me to have been but imperfectly understood, I have drawn up the following short sketch of the principal forms of sloughing sore, and the treatment which experience has led me to adopt in each.

The singular discordance which exists in the treatment proposed by different authorities in these diseases, might naturally lead to the conclusion, that cases very different in their nature had been confounded under the single appellation of sloughing sore. This I believe to be true. The various plans of treatment which have been recommended, by bleeding and diaphoretics, by tonics, or by mercury, are all judicious and beneficial in some cases, and all injurious in others, and the difference of the effect may be accounted for by some differences in the character of the disease. The distinction, I apprehend, will be found to be in general exceedingly simple, and to consist chiefly in the state of the capillary circulation in the immediate vicinity of the sore. In a part which is sloughing, the circulation may be either above or below the natural level, and it is the duty of the surgeon to reduce it where it is excessive, and to raise and excite it where it is deficient in activity and tone. The appearance of the edges and surrounding skin will, I believe, be found to be a far more accurate and delicate criterion of the state of the circulation in the part, than either the state of the pulse or the temperature of the skin; and, by a due attention to this circumstance, the practitioner will seldom have reason to change his plan of treatment, and will very rarely be disappointed in the ultimate result.

On these principles the following division has been made, which will, I believe, be found to comprehend all the more important varieties.

1. Perhaps the most common form of sloughing sore is that which was exemplified in the case of Ann Collins.* In men, it is found usually on the prepuce. The mode of its commencement varies. Occasionally the sloughing attacks a sore possessing the usual characters of what is called the Hunterian chancre; but more commonly the first appearance is as a small swelling, or lump, of the size of a large pea, not marked by any circumstances which are calculated to excite particular attention. In the course of a day or two, there appears in the centre of this swelling a small yellow hole, which enlarges rapidly, soon becoming black, and assuming the following appearance :—The whole aspect is that of acute inflammation; the surface of the sore, as has been said, is dark; the surrounding parts are much swelled, and of a *bright arterial* colour. The swelling is of that kind which usually marks acute inflammation; that is, there is deposition of lymph in the cellular membrane, as well as effusion of serum. There is, however, not commonly any distinct indurated base, since, if such induration exists in the earlier stages of the sore, it is rapidly destroyed by the spreading of the slough, and, unless the progress be unusually slow, there is no time for its re-formation. The pain is severe, but not more so than is readily accounted for from the violence of the inflammation. The general system almost always sympathises with the local disorder, being affected with the ordinary marks of inflammatory action. The more plethoric the temperament of the patient, the more rapid is the progress of the sloughing.

By bleeding, antimony, diaphoretics, and purgatives, the inflammation may be subdued, and the sloughing arrested. The sore will then sometimes granulate and heal; but more

* See No. 3, (Sept. 1826,) page 204.

commonly the improvement under this treatment is only temporary, and the sloughing recurs in successive attacks until mercury is employed. Thenceforward the recovery is easy and uninterrupted. If the surgeon can trust with confidence to his own judgment, the mercury may be employed in the first instance, either with or without the other means which have been mentioned. Indeed, if the progress is not very rapid, the cure may be trusted, as in the case of Ann Collins, to mercury alone. Mercury will depress arterial action with no less certainty than antimony, and these depressing effects are no sooner felt in the system, than the skin surrounding the ulcer loses its bright florid hue, the tumefaction subsides, and the sloughing ceases to spread. This sore presents no difficulty. The effect of the remedies is almost always uniform, and nothing but an unfounded dread of mercury on the part of the surgeon can perplex the treatment, or affect the facility of the cure. It may be assumed as a principle, that, as long as acute inflammation exists, mercury never either occasions or aggravates sloughing.

2. Another form of sloughing sore, much less common than the preceding, but far more formidable, is that of which an instance was given in the case of John Bourke.* It is chiefly met with in the groin, but sometimes also on the penis. The state of the parts is exactly opposite to that which prevails in the first species. There is little pain, and no tumefaction. The edge is not everted or elevated, but is on a level with the surrounding integuments. It is encircled by a narrow border of *very faint pale-coloured* inflammation, fading, at the distance of half an inch or an inch, into the colour of the neighbouring skin, which is more than usually white, and as it were bloodless. The slough does not generally form in large black masses, or separate in distinct portions, but appears as a yellow fringe attached to the surrounding skin, and hanging loose and flaccid into the profuse discharge which fills the sore. As a successive edging of slough forms, the original portion becomes involved in the discharge and is lost, much as the edge of a piece of melting lead gradually sinks and is lost in the fused metal. The ulceration is often most extensive, but it chiefly involves the skin and the adipose membrane, frequently dissecting, but rarely destroying, the muscles. The state of the pulse varies. It is generally deficient in strength, though it is most commonly frequent and full, and is easily excited by stimulants of any kind.

But the surgeon cannot be too strongly cautioned against

* See No. 3, (Sept. 1826,) page 207.

trusting to inferences drawn from the pulse or the skin, in preference to those which are suggested by the edge of the ulcer; the appearance of which affords convincing proof of the languor of the circulation in the part, and sufficiently indicates the employment of tonics. Bark, quinine, mineral acids, and sarsaparilla, are the appropriate remedies, and, if steadily continued, will generally, even in aggravated cases, succeed in arresting the disorder. But, if the surgeon, wearied by the obstinacy of the case, and alarmed at the imminent danger of the patient,—doubtful also, perhaps, of the justice of his original opinion, and recollecting the benefit which he has witnessed in other cases from depletion or mercury, should resort to either of these remedies in the present instance, the error is always injurious, and frequently fatal. The existing atony of the blood-vessels is often so far increased, that the progress of the sloughing cannot afterwards be stopped, and the malady not unfrequently terminates in death.

In these cases hemorrhage is by no means an uncommon occurrence. This is an accident to which all sloughing sores are liable, but it is not in all equally dangerous. In the first species it frequently occurs to a profuse and formidable extent, from the force of the circulation combined with the violence of the sloughing; but I have never seen any instance where serious consequences ensued. The loss of blood relieves the inflammation and checks the disease, and the hemorrhage is not subject often to recur. But, in this second form of sore, the case is altogether different. Large bleedings will return at considerable intervals, and often so reduce the strength of the patient as most seriously to aggravate his danger. It is sometimes supposed that the hemorrhage takes place after the sloughing has been stopped, and on the separation of the eschar; but this I apprehend is an error. On the contrary, it is at the moment when the sphacelation is proceeding with the greatest rapidity that this consequence is chiefly to be apprehended. It is most probable that the process of obliteration of the blood-vessels by the effusion of lymph, which is known to be usually carried on in the neighbourhood of a sphacelated part, does not in these instances take place, in consequence of the debility of the system, so that, when the coats of the yet pervious arteries become involved in the slough, the blood forces its way through the flaccid and corrupted mass. At any rate, no means of preventing the return of the hemorrhage will be found so effectual as the continued use of tonics, by which the blood-vessels are gradually restored to their natural health and vigour.

3. There is a third species of sore, which differs from the preceding rather in its external appearance than in its essential characters. It seldom occurs unless active inflammation has preceded, and frequently follows the copious use of mercury at some previous period. There is considerable tumefaction. The colour of the circumference, instead of being bright and florid, is of a *deep venous red,* often inclining to purple; and marks rather the stagnation of the blood in the enlarged and weakened vessels, than any increased vigour or increased activity of circulation. The slough is usually of a dark hue. There is little pain attending this form of sore, nor is the progress rapid; but it is extremely obstinate and difficult of cure. Tonics are as necessary here as in the last species, and the danger from mercury is equally great. If this remedy is freely exhibited in the advanced stages of the disease, it not unfrequently happens that the penis, the scrotum, and the testicles, and sometimes the life itself, fall a sacrifice.

4. In the forms of sore which have been noticed, the principle of treatment is simple, and the diagnosis easy. The colour and state of the surrounding integuments afford a test which will seldom leave the surgeon in uncertainty, and never mislead him; and the treatment is not often liable to interruption from any change in the state of the disorder. But there is another sore on the penis, by no means uncommon, of which the description is more difficult, and the treatment more complicated. It is properly rather a foul than a sloughing ulcer, but, as it is liable to sudden and violent attacks of sloughing, it demands a place in this enumeration.

It may occur on the glans or at the meatus urinarius, but its most usual seat is behind the corona glandis, spreading upwards on the body of the penis, between the corpora cavernosa and the præputium, and dissecting the one from the other. The surrounding parts are in a state of active, but not of acute inflammation. The colour of the prepuce is florid, and it is generally swelled and œdematous, but so little thickened or rigid that the œdema is obviously the only obstacle to its complete retraction. There is hardness, but it does not extend beyond the base and the edges of the sore, and is indeed nearly confined to the spreading edge; for it is of the nature of the sore to heal at one part and to spread at another. When the sore has burrowed between the corpora cavernosa and the prepuce, this thickened edge can be felt externally, and usually it forms a hard ring encircling the penis, marking the extent of the ulceration, and creeping nearer and nearer to the pubes as the disease proceeds upwards. The discharge is brown, watery, and copious. The pain is not very severe, but there is much constitutional disturbance, a quick and irritable pulse, and some heat of the skin. As long as this sore proceeds by ulceration, its progress is very slow; but in the sudden attacks of sloughing, to which it is subject, large portions either of the penis or the prepuce, but generally of the former, are frequently destroyed. These devastations fall sometimes on the glans, but chiefly on the corpus spongiosum; so that there are few advanced cases in which a hole has not in this way been opened into the urethra, about half an inch or an inch above the situation of the frænum. Until the discharge assumes a healthy appearance, and the thickened edge subsides, any apparent improvement is only deceptive.

It is the liability to slough which forms one of the distinguishing characters of this sore, and constitutes the principal difficulty in treating it. The state of the surrounding parts sufficiently shows that there is active, though not violent inflammation, and indicates the employment of antiphlogistic remedies. But there is little real tone; and, if this treatment be carried to any extent, the parts pass into the opposite extreme, and extensive sloughing ensues. Mercury is, of all remedies, the most beneficial, and seldom fails at first to lessen the inflammation, to correct the discharge, to reduce the thickening, to clean the surface of the sore, and to cause a commencement of cicatrisation. But if the remedy is continued and pushed, so as to produce the usual depressing effects of mercury, the consequence is, almost invariably, that the prepuce acquires a dark colour, and the sore sloughs in the violent manner which has been already described: and this effect can only be avoided by conducting the mercurial course with the greatest caution, and combining with it sarsaparilla or some other tonic, which is capable of supporting the system during its continuance. As the secondary symptoms which follow this sore are peculiarly obstinate and dangerous, and can only, I believe, be escaped by the early cure of the primary ulcer, too much attention cannot be paid to the regularity and management of the mercurial course.

5. There is yet a fifth species of sloughing sore, which is perfectly distinct from any of the preceding, and an instance of which was given in the case of Sarah Starkey.* It is not common: I never saw it except in women, nor except in a hospital. In the Lock Hospital it occurs two or three times in the course of the year, and I believe the proportion has always been about the same. It is almost confined to the lowest order of prostitutes, who have been subject to great hardships, and have been in the habit of drinking spirits

* See No. 3, (Sept. 1826,) page 205.

largely. I have never been able to trace it to any particular part of the town; nor do I believe it to depend on infection, as in no case have I seen it followed by secondary symptoms, though in the Lock Hospital it has never, in my recollection, been treated with mercury. Indeed, I have seen it originate in a patient who had been for some time an inmate of that institution, where it has long been known under the just, though not very distinctive, appellation of Constitutional Sore.

This sore is excessively painful. It may be situated on any part of the labia, perineum, nates, or thighs in their neighbourhood. The surface is covered with a thick dark slough; the discharge is thin and ichorous; the edges are tumid and partially everted; the circumference, to a considerable extent, is violently inflamed, but of a *dark-red* colour. It is usually circular, or nearly so, and spreads most rapidly and extensively in every direction. The pulse is greatly accelerated during the progress of the sloughing, but there is not much heat of the skin, or appearance of tone about the system. The pain is most excruciating and constant, and its severity is proportioned to the rapidity of the sphacelation. This ulcer is sufficiently distinguished from the first species by the dark colour of the surrounding integuments, and from the third species by the extraordinary violence of the pain, and the extent and rapidity of the destruction which it occasions.

This sore never resists full doses of opium. Until the pain is subdued, no benefit whatever is discernible; but, as soon as this effect is produced, the sloughing is arrested. There is consequently no limit to the dose in which opium should be exhibited, until full relief is experienced; and, in order to accelerate this object, the external application of it should be combined with its internal use. Opium is useful in all cases of painful sloughing, but, in other cases of sloughing sores on the genitals, the benefit is for the most part temporary: in this it is permanent. In the greater number of instances the sloughing does not recur, and the sore readily cicatrises; the period of recovery being proportioned to the extent of the destruction. Sometimes, as in the case of Starkey, it is necessary to combine with the opium some mild tonic.

I apprehend that it is in this sore that such remarkable advantage has been derived from the application of the concentrated nitric acid. The pain is in the part which is undergoing sphacelation, and if the process can be brought at once to a termination by means of a caustic, the relief is as complete as from the use of opium. In whatever way relief is obtained, the effect on the progress of the sore is equally certain.

In the preceding very brief sketch of the general plans of treatment which I have been led to adopt in these diseases, I have confined myself to the result of my own experience,—not assuredly from any conviction of the superiority of the remedies which I have used over those which have been employed by other surgeons, but from my inability to analise in the same way the principles on which their practice is founded. There are doubtless some circumstances in the foregoing statement which a more extensive experience will correct, and many which it will render clear and distinct; but, of the justice of the general distinctions I can entertain no doubt. They are neither novel nor complicated; but I am persuaded that every day's experience will show that they are not on that account the less practically important, and that they cannot be neglected without subjecting the patient to all the hazard of an uncertain empiricism.

*Golden-square; August*, 1827.

